# Deep learning quantification of vascular pharmacokinetic parameters in mouse brain tumor models

**DOI:** 10.31083/j.fbl2703099

**Published:** 2022-03-16

**Authors:** Chad A. Arledge, Deeksha M. Sankepalle, William N. Crowe, Yang Liu, Lulu Wang, Dawen Zhao

**Affiliations:** 1 Department of Biomedical Engineering, Wake Forest School of Medicine, Winston-Salem, NC 27157, USA; 2 Department of Cancer Biology, Wake Forest School of Medicine, Winston-Salem, NC 27157, USA

**Keywords:** convolutional neural network, dynamic contrast enhanced MRI, pharmacokinetic modeling, glioblastoma, breast cancer brain metastasis, vascular permeability parameters

## Abstract

**Background:**

Dynamic contrast-enhanced (DCE) MRI is widely used to assess vascular perfusion and permeability in cancer. In small animal applications, conventional modeling of pharmacokinetic (PK) parameters from DCE MRI images is complex and time consuming. This study is aimed at developing a deep learning approach to fully automate the generation of kinetic parameter maps, Ktrans (volume transfer coefficient) and Vp (blood plasma volume ratio), as a potential surrogate to conventional PK modeling in mouse brain tumor models based on DCE MRI.

**Methods:**

Using a 7T MRI, DCE MRI was conducted in U87 glioma xenografts growing orthotopically in nude mice. Vascular permeability Ktrans and Vp maps were generated using the classical Tofts model as well as the extended-Tofts model. These vascular permeability maps were then processed as target images to a twenty-four layer convolutional neural network (CNN). The CNN was trained on T_1_-weighted DCE images as source images and designed with parallel dual pathways to capture multiscale features. Furthermore, we performed a transfer study of this glioma trained CNN on a breast cancer brain metastasis (BCBM) mouse model to assess the potential of the network for alternative brain tumors.

**Results:**

Our data showed a good match for both Ktrans and Vp maps generated between the target PK parameter maps and the respective CNN maps for gliomas. Pixel-by-pixel analysis revealed intratumoral heterogeneous permeability, which was consistent between the CNN and PK models. The utility of the deep learning approach was further demonstrated in the transfer study of BCBM.

**Conclusions:**

Because of its rapid and accurate estimation of vascular PK parameters directly from the DCE dynamic images without complex mathematical modeling, the deep learning approach can serve as an efficient tool to assess tumor vascular permeability to facilitate small animal brain tumor research.

## 1. Introduction

Glioblastoma multiforme (GBM) is the most common and lethal primary brain cancer. GBM is characterized by invasive tumor cell growth and extensive angiogenesis. Despite the development of highly angiogenic and leaky microvessels, many GBM cells grow by co-opting the pre-existing vessels, where the blood-brain barrier (BBB) may remain intact. Thus, the BBB disruption in GBM is markedly heterogeneous, which prevents therapeutic concentrations of chemotherapeutic agents from reaching the tumor in brain parenchyma [1–5].

MRI provides a noninvasive tool for acquiring information about the tumor microenvironment. Advancements in MR techniques have increased noninvasive access to a significant amount of useful information on cancer metabolism and tumor heterogeneity, ranging from spatial scaled to functional or metabolic imaging. Dynamic contrast-enhanced (DCE) MRI is a widely used technique for obtaining information of vascular perfusion and permeability [6–10], A series of T_1_-weighted (T_1_-w) images is acquired before and after an i.v. bolus injection of gadolinium chelates to capture the transport of the contrast agent (CA) into and away from a tissue/region. By measuring dynamic changes in T_1_-w signal intensity (SI) as a function of time and applying pharmacokinetic (PK) modeling, exchange of the CA between the intravascular and extravascular extracellular space (EES) can be quantitatively estimated to extract several vascular perfusion/permeability parameters. These parameters include the volume transfer coefficient (Ktrans), flux rate constant (Kep), fractional volume of the blood plasma (Vp), and fractional volume of the EES (Ve) [6,7,11]. However, PK modeling of gadolinium in DCE MRI is complex. Various PK models have been established including the Brix model [12], the classical Tofts model [13,14], and the extended Tofts (Ex-Tofts) model [15]. The Tofts model is widely used to estimate Ktrans, Kep, and/or Ve. The Ex-Tofts model is an extension upon the Tofts model that takes into account the volume of contrast in the blood plasma, Vp, in addition to Ktrans, Kep and Ve.

PK mathematical models require knowledge of the arterial input function (AIF), which is an important variable for the modeling accuracy [16–20]. Lack of accurate measurement of the AIF, also know was the vascular input function (VIF), represents a major disadvantage of current PK models as a small deviation in the AIF can markedly affect the estimation accuracy of kinetic parameters. Measurements of the AIF is additionally more difficult if not impossible in small animals in a case-by-case basis. Hence, a population-based averaged AIF from literature assuming a consistent value is implemented here to avoid poor AIF manual measurements leading to poor PK parameter accuracy. Population-based averaged AIFs have additionally been shown to improve the reproducibility of PK parameters [18,20]. Moreover, PK models are solely based on fitting pixel parameters to the CA concentration curves [21]. The pixel-wise model fitting is computationally demanding for a whole MR scan with thousands of pixels and thus is commonly time and memory consuming. Furthermore, manual identification of CA arrival during the dynamic scans as well as additional MRI acquisitions for T_1_ measurements are also required for accurate modeling of PK parameters when using PK models.

To address these limitations, we are seeking a machine learning approach for faster DCE parameter estimation without human bias. Machine learning has been increasingly used to power various research areas as it provides us with the ability to augment the knowledge and efforts of experts with a tool that can help to analyze data, and consequently make better predictions and decisions. Machine learning is commonly divided into two general forms, unsupervised and supervised learning. Unsupervised learning is concerned with understanding the internal structures of unlabeled data in a fully automated manner. Supervised learning requires training data to be manually labeled, where the algorithm learns from the labeled training data to predict the correct label for the unlabeled test data [22]. The model developed in this study is a supervised deep learning algorithm. Compared to the conventional machine learning methods, which require considerable research and design effort to capture higher-level features of the data, deep learning models learn data representations at various levels of abstraction automatically [23,24]. One such advanced deep learning algorithm used for images is the convolutional neural network (CNN).

**Fig. 1.**
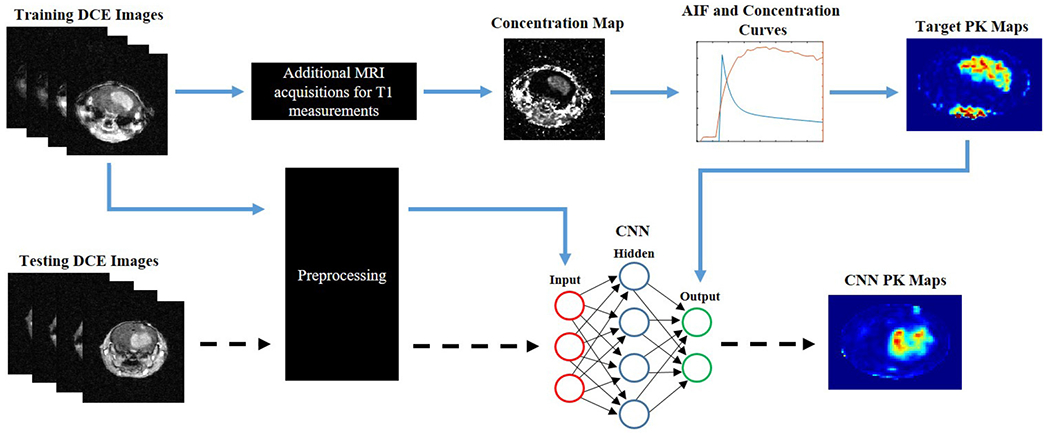
Pipeline and workflow of this project. Conventional PK modeling is applied to DCE dynamic images to generate target PK maps. Source DCE dynamic images and target PK maps are fed into the 24-layer CNN for training (blue arrows). An independent testing DCE dynamic imaging dataset can be fed directly into the CNN to generate CNN PK maps following training of the neural network (dashed arrows). Preprocessing of the DCE dynamic images involved reshaping, segmentation, and batch normalization. The neural network can directly predict the PK maps with the DCE time series alone. In contrast, conventional PK modeling requires manual CA arrival identification, additional MRI acquisitions for T1 measurements, complex curve-fitting, as well as the knowledge of the AIF along with the DCE time series in order to estimate these kinetic parameters.

Recent studies have sought implementation of deep learning to facilitate clinical PK parameter generation [25–27]. Nalepa *et al*. [26] have developed a clinical end-to-end DCE processing pipeline for brain tumors. However, this approach uses deep learning only for tumor segmentation and hence still requires conventional PK modeling. Choi *el al*. [25] implemented deep learning to improve the reliability of the AIF generation in astrocytomas to improve PK parameter accuracy; however, similar to Nalepa *et al*. [26], this approach still requires conventional PK modeling. In an effort to develop a possible surrogate to conventional PK modeling, Ulas *et al*. [27] have demonstrated the feasibility of implementation of deep learning for automatic PK parameter generation directly from T_1_-w DCE images in stroke patients. These studies have highlighted the potential of deep learning to facilitate clinical PK parameter generation; however, to the best of our knowledge there have been no efforts to apply these approaches for small animal brain tumor studies. Similar to the methodology proposed by Ulas *et al*. [27], we have developed a deep learning-based CNN to directly estimate the vascular parameters in brain tumors derived from DCE MRI without conventional PK modeling aimed at facilitating small animal glioma research (Fig. 1, Supplementary Fig. 1). The developed CNN in this project contains two pathways to efficiently capture multiscale features. The local pathway of the CNN extracts the details of local image regions in the filter window, whereas the global pathway focuses on determining the contextual and structural information. Due to its strong generalization ability, this deep learning algorithm shows increased robustness to signal noise and can suppress any non-consequential parameters. Moreover, this CNN method estimates the kinetic parameters directly from the SI curve of CA over time. This eliminates several intermediate steps including manual CA arrival identification, additional MRI acquisitions for T_1_ measurements, estimation of the AIF, and application of curve-fitting, thereby, accelerating the process and avoiding human bias (Fig. 1).

## 2. Materials and methods

### 2.1 Orthotopic GBM and breast cancer brain metastasis mouse models

All animal procedures performed were approved by the Wake Forest University Institutional Animal Care and Use Committee. Human glioblastoma U87 cells were cultured in Dulbecco’s modified Eagle’s medium (DMEM) with 10% FBS, 1% L-Glutamine, and 1% penicillin-streptomycin at 37 °C with 5% CO_2_. Once 80% confluence was achieved, the cells were harvested and suspended in serum-free medium. U87 cell suspensions (1.2 × 10^4^ cells in 4 *μ*L serum-free medium) were injected intracranially to the right caudal diencephalon of nude mice (n = 6) [28–30].

A breast cancer brain metastasis (BCBM) mouse model with intracardiac injection of brain tropic breast cancer cells has been previously established at the lab [31,32]. Briefly, MDA-MB-231-BR cells (kindly provided by Dr. Steeg, NCI) were cultured in DMEM with 10% FBS, 1% L-Glutamine, and 1% penicillin-streptomycin at 37 °C with 5% CO_2_. Once 80% confluence was achieved, the cells were harvested and suspended in serum-free medium. 2 × 10^5^ MDA-MB-231-BR cells in 50 *μ*L serum-free medium were injected directly into the left ventricle of nude mice (n = 3) under ultrasound imaging guidance (Vevo LAZR, FUJIFILM VisualSonics, Inc. Toronto, Canada).

### 2.2 MRI acquisition and DCE MRI

Two weeks after tumor implantation, MRI was conducted. All MR imaging was performed on a Bruker 7T Biospec 70/30 USR scanner (Bruker Biospin, Rheinstetten, Germany). The mice were anesthetized in a chamber with 3% isoflurane, placed in a 30 cm horizontal bore magnet, and the tail vein was catheterized for bolus injection of Gd-DTPA (Magnevist; Bayer Healthcare) using a 27G butterfly. Animal respiration was monitored and maintained throughout MRI experimentation using a respiratory bulb. Anatomical T2-w images were acquired using a Rapid Imaging with Refocused Echoes (RARE) sequence (TR/TE: 2500/50 msec; Number of Scan Averages (NSA): 8; Echo Train Length (ETL): 8, Field of View (FOV): 22 mm × 22 mm; Slice Thickness (ST): 1 mm; scan time: 5 min 22 sec). Before DCE-MRI, high-resolution images were captured for T_1_ mapping by variable flip angles (TR/TE: 100/2.24 msec; NSA: 6; FOV: 22 mm × 22 mm; ST: 1 mm; Flip Angle (FA): 5, 10, 20, 35 degrees; scan time: 57 sec/FA). DCE-MRI was performed using a rapid T_1_-w FLASH image sequence (TR/TE: 43/2.3 msec; FA: 30 degrees; FOV: 22 mm × 22 mm; NSA: 2; scan time: 6 min 57 sec) with dynamic images acquired before and after the bolus injection of Gd-DTPA (0.1 mmol/kg, i.v.) to ensure capture of Gd-DTPA kinetics. For each animal, dynamic images of five consecutive slices were acquired were to cover the tumor region [32]. Lastly, T_1_-w contrast enhanced imaging was performed using a T_1_-w RARE sequence (TR/TE: 800/7 msec; ETL: 8; NSA: 8; FOV: 22 mm × 22 mm; scan time 2 min 33 sec). All anatomical imaging covered the region from the frontal lobe to the posterior fossa.

The acquired images were extracted in both raw Bruker 2dseq and DICOM file formats. An additional 3D Gaussian filter was used on DCE dynamic data. DCE images and variable flip angle T_1_ series were co-registered with T2-w images to eliminate motion artifacts due to respiration. Homemade MATLAB R2020b scripts were developed for data processing and storage.

### 2.3 Construction of DCE MRI permeability parameters using PK models

Co-registered T_1_ multiple flip angle images that were acquired at angles 5, 10, 20, and 35 were used to calculate T_10_ maps using the method proposed in Brookes *et al*. [33]. The SI of each flip angle image is stacked into an array for each pixel. X coordinates were computed by dividing the SI at each pixel by tangent of the respective variable flip angle (VA).


(1) $$X=\frac{SI}{\tan(VA)}$$

Similarly, Y coordinates were calculated by dividing the SI by sine of each angle.


(2) $$Y=\frac{SI}{\sin(VA)}$$

These coordinates were then linearly fitted to quantity the slope (Sp). The T_10_ values, tissue relaxation precontrast, at each pixel is estimated by:

(3) $$T_{10}=-Tr/\log(Sp)$$

where Tr is the repetition time of the MR scan. These T_1_ maps were used to generate CA concentration maps based on Brookes *et al*. [33] from the motion-corrected dynamic images. Dynamic SI was used to evaluate the CA concentration at each pixel. The equations involved were:

(4) $$\frac{1}{T_{1}}=\frac{1}{T_{10}}+r_{1}*C_{t}(t)$$

where T_1_ is deducted from T_10_ and which includes the presence of CA. r_1_ is the relaxivity, which is 3.11 s^−1^ mM^−1^ for Gd-DPTA at 7T. The final C*_t_*(t) was determined from S(t), the SI of the dynamic time series, given by

(5) $$S(t)=S_{0}\frac{\left(1-e^{-T_{r}/T_{1}}\right)\mathrm{Sin}\theta }{1-e^{-T_{r}/T_{1}\mathrm{Cos}\theta }}$$

where S_0_ is the initial single intensity of the dynamic time series at each pixel. *θ* is the flip angle of the DCE MRI sequence. A population-based averaged AIF determined from literature was implemented to avoid poor AIF estimation and to enhance the reproducibility of PK parameters [18,20]. Here a bi-exponential AIF was implemented as AIFs fit well to a bi-exponential decay model as suggested by Cheng *et al*. [17]. The AIF was generated according to Eqn. 6.


(6) $$AIF(t)=ae^{-c_{1}t}+be^{-c_{2}t}$$

where a and b are the coefficients determined from a population average of the AIF and c_1_ and c_2_ are the rates of decay of the AIF. c_1_ and c_2_ represent the uptake and washout of CA respectively. The Tofts model, Eqn. 7, and the Ex-Tofts model, Eqn. 8, were each applied to dynamic DCE data to estimate the kinetic parameters from CA concentration in the tissue and the AIF.


(7) $$C_{t}(t)=K_{\text{trans}}\int_{0}^{t}C_{p}(t)e^{-K_{ep}(t-\tau )}d\tau$$

(8) $$C_{t}(t)=V_{p}C_{p}(t)+K_{trans}\int_{0}^{t}C_{p}(t)e^{-K_{ep}(t-\tau )}d\tau$$

where Ktrans is the transfer rate constant from the blood plasma to the EES, Kep is the transfer rate constant from the EES to the blood plasma, and Vp is the fractional volume of blood plasma. C*_t_*(t) is the concentration of CA in the tissue and C*_p_*(t) is the concentration of CA in blood plasma (AIF) [13,14]. Ve is the fractional EES volume that is the ratio of Ktrans to Kep. Here Tofts Ktrans, Ex-Tofts Ktrans, and Ex-Tofts Vp maps were generated and used for training and testing a convolutional neural network.

### 2.4 Convolutional neural network

In this study, the parameters are considered as a mapping problem between the dynamic time series and parameter maps. The CNN is trained to map the concentration of CA to the output. The estimated parameters from either the Tofts model or the Ex-Tofts model are processed as target images to the 24-layer CNN (Supplementary Fig. 1). The first convolutional layer applies 2D filters to each channel individually to extract low-level features. This was then followed by the two parallel pathways, local and global. The global pathway consists of 3 dilated convolutional layers each followed by a ReLU activation layer. The layers are dilated by factors of 2, 4 and 8, which captures the increase in receptive field size. This preserves the global structure and profile of the image. The local pathway consists of 3 non-dilated layers each of which are also followed by a ReLU activation layer. ReLU is applied to initiate nonlinearity into the mapping.

Zero-padding is applied to every convolutional operation to keep the spatial dimensions of the output the same as the input. The filter size of each convolutional layer is 4 × 4. The first convolutional layer has 128 filters, whose feature maps are then fed into the dual pathways discussed above. Each layer in both the global and local pathways has 128 filters, which produce 128 × 128 × 128 feature maps each at the end. The pathways are concatenated to form a multiscale feature set. Following this, 4 fully connected layers of 1024, 512, 128 and 1 hidden node are used to derive the best feature combination that can accurately predict the outcome. Each convolutional layer was followed by a ReLU layer except for the last convolutional layer with one hidden node. Finally, a regression output layer with 128 × 128 × 1 node estimates the output maps. To maintain the spatial dimensions of the images, a regular convolution layer with 1 × 1 filters is considered to be a fully connected layer.

The raw data from DCE MRI for each GBM animal was reshaped to have the temporal series as the third dimension and scan slice as the fourth dimension. Data sets were resized to 128 × 128 × 40 × 5 by removing sequential images of the DCE scans involving more than 40 dynamic images. Each slice from the six animals were then stacked to create a single four-dimensional data set of size 128 × 128 × 40 × 30. Both the DCE dynamic images as well as the PK target maps were segmented to remove the skull and peripheral tissue surrounding the brain. DCE MRI data sets were batch normalized in each plane before feeding them into the network [34]. Training was performed using k-fold cross validation. To this end, imaging datasets from one mouse were isolated and held out for future testing. A neural network was then trained on the remaining five mice (n = 25 slices) and was then tested with this isolated imaging dataset (n = 5 slices). This process was then repeated de novo for a total of six times allowing each animal to serve as the testing data in separately trained networks. As we generated three separate PK target maps (Tofts Ktrans, Ex-Tofts Ktrans, and Ex-Tofts Vp), a total of 18 networks were trained. Among the image datasets left following isolation of the testing case, 80% were selected for training and 20% for validation. Selection of the validation data from training datasets was performed by sorting out a single random slice from each of the remaining five mice for each network. This was randomized for each of the networks to allow for rotation through the validation data in addition to rotation through training and testing data.

The networks were trained with a learning rate of 10^−4^ and an L2-regularization of 10^−4^ using the Adam optimizer [35], A maximum of 600 epochs for a minibatch size of 20 was determined to prevent poor generalization when the validation loss stops improving. The validation checks were scheduled for every 2 iterations of training. Once each network was trained and the network parameters were determined, the test data set of the dynamic time series was fed into the network to directly predict the kinetic parameters. All of the code was implemented on MATLAB using the deep learning toolkit and an imported Keras library on a 3.7 GHz processor with 16 GB RAM. Each training session required an average time of 380 minutes.

A transfer study was then performed to assess if the GBM trained CNN could accurately predict PK maps for alternative brain tumors. Here, a total of 20 brain lesions detected in the three BCBM mice were implemented for testing. The DCE images of BCBM were preprocessed similar to the DCE images from the GBM study: reshaping of dynamic images to 128 × 128 × 40 × 5, segmentation to remove the skull and peripheral tissue surrounding the brain, and batch normalization. All six GBM mice (n = 30 slices) were used as training data and fed into the aforementioned 24-layer CNN and trained using the same network parameters as the GBM study. A total of three networks were trained, one for each PK parameter (Tofts Ktrans, Ex-Tofts Ktrans, and Ex-Tofts Vp). Once the networks were trained and the network parameters were determined, the preprocessed test data set of the dynamic time series for each brain metastasis case were fed into the network to directly predict the kinetic parameters.

### 2.5 Image processing

All image processing was performed in MATLAB using homemade scripts. Following testing of the proposed CNN, an ensemble of all networks was performed for each study. Intratumoral PK parameters from both the target PK parameter maps and the respective CNN maps were isolated for pixel-by-pixel comparisons. To this end, anatomical co-registered T_1_-CE images were used to manually mask the enhancing lesions and applied to corresponding PK parameter maps. For those that were non-enhancing on T_1_-CE images in the BCBM transfer study, co-registered T2-w images were used. Peripheral GBM tissue was defined as a single pixel towards the center of the lesions from the outer T_1_-CE lesion masks.

### 2.6 Statistical analysis

Statistical analysis was performed using Microsoft Excel (Microsoft Corporation, Redmond, WA, USA) and GraphPad Prism 9.0 (GraphPad Software, San Diego, CA, USA). Pixel-by-pixel comparisons of Ktrans and Vp values within tumor regions between the CNN and the PK models were conducted. Linear regression was applied to provide statistical correlation and significance. To compare the similarity between the CNN maps and the corresponding PK target maps, root mean squared error (RMSE) values of the brain tumor were calculated along with normalized RMSE (nRMSE) values based on target PK parameter standard distributions (SD). Bland-Altman plots were generated to assess general trends in the predictive capability of the proposed CNN.

## 3. Results

**Fig. 2.**
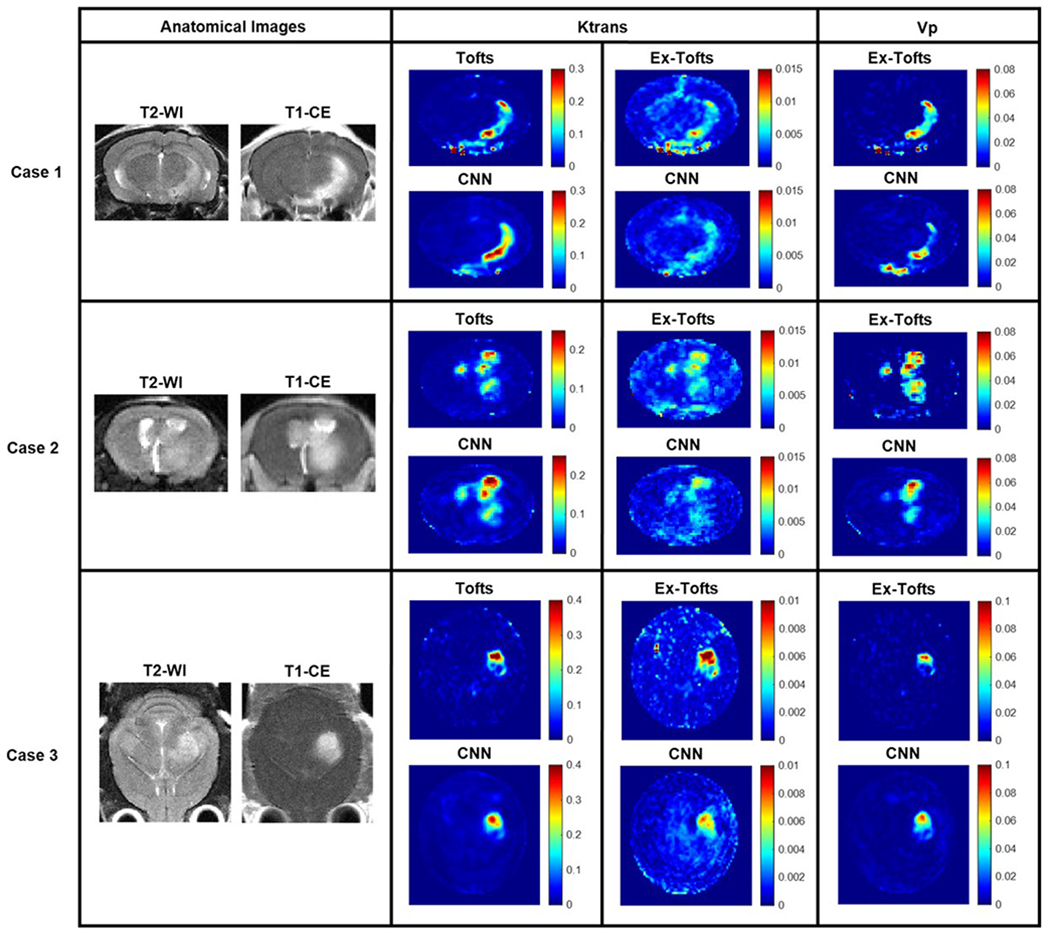
GBM study. Three cases from the GBM study with permeable lesions. Anatomical T2-w and T_1_-CE images were provided for reference. Tofts Ktrans, Ex-Tofts Ktrans, and Ex-Tofts Vp target maps as well as their corresponding CNN PK maps all displayed intratumoral heterogeneity and increased permeability.

The Tofts Ktrans, Ex-Tofts Ktrans, and Ex-Tofts Vp generated maps were used as target maps to train the deep neural network. The time required to generate maps by the trained CNN was less than a few seconds with no human interference or additional data. The Tofts and Ex-Tofts model required additional MRI acquisitions for T_1_ mapping thus increasing scan times and took longer to generate PK maps (Supplementary Table 1). In particular, the Ex-Tofts model typically took times up to five minutes for parameter map generation due to the need for non-linear least squares curve-fitting. These PK models necessitated additional data of CA arrival, T_1_ mapping, complex curve-fitting algorithms, and knowledge of the AIF. For the k-fold cross validation GBM study, RMSE and nRMSE intratumoral values were calculated for each network to assess the performance of the proposed neural network across each network iteration (Table 1). As shown in Fig. 2, GBM lesions were identified on anatomic T2-w and T_1_-CE images. The hyperintense signal on T_1_-CE images resulted from high permeability of tumor vasculature. These tumors were used as testing cases in separately trained networks. Target maps of Tofts Ktrans, Ex-Tofts Ktrans, and Ex-Tofts Vp all exhibited intratumoral heterogeneity and high permeability parameter values in the tumor region. Clearly, the corresponding CNN PK maps recapitulated this trend of intratumoral heterogeneity and increased permeability. Moreover, we applied a network trained solely with axial images to a coronal imaging dataset (Fig. 2, Case 3). The CNN successfully recapitulated coronal target PK parameter maps, suggesting this CNN approach is image orientation-independent.

**Fig. 3.**
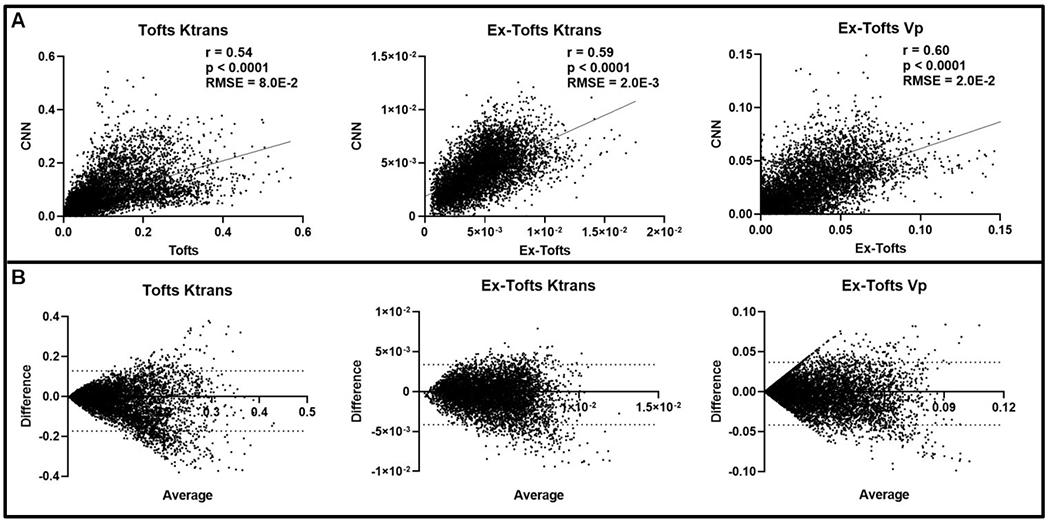
Intratumoral ensemble analysis of GBM. (A) Pixel-by-pixel data (n = 7606) of intratumoral regions were plotted, revealing a significant linear correlation for Ktrans and Vp between CNN and target PK models (*p* < 0.0001). Low RMSEs were found between the target PK models and the CNN for all three parameters (RMSE < Target PK parameter SD). (B) Bland-Altman plots of PK parameters were generated with dashed lines corresponding to the upper and lower 95% confidence intervals.

To investigate if the CNN approach can decipher intratumoral heterogeneity, we conducted pixel-by-pixel comparisons between the CNN and the target PK models. Only the tumor region was considered following an ensemble of all networks for the GBM study (n = 30 slices), containing a total of 7606 pixels. A significant linear correlation (*p* < 0.0001) was found for each of the three target PK parameters and their corresponding CNN PK values (Fig. 3A). Similarly, all three PK parameters displayed low RMSE values less than the SD of the target PK parameters, indicating the ability of the CNN to generate PK values with minimal error. Furthermore, Bland-Altman plots indicated that the CNN did not show any trends in over-predicting or under-predicting the anticipated PK parameter values (Fig. 3B). We further investigated the predictive capabilities of the CNN for peripheral tumor tissue alone. Similar to the whole tumor region, a significant linear correlation (*p* < 0.0001, n = 2236) was found for each of the three target PK parameters and their corresponding CNN PK values (Supplementary Fig. 2A). RMSE values less than the SD was found for all PK parameters. Bland-Altman plots indicated that the CNN showed negligent over-predicting or under-predicting of the anticipated PK parameter values (Supplementary Fig. 2B).

**Fig. 4.**
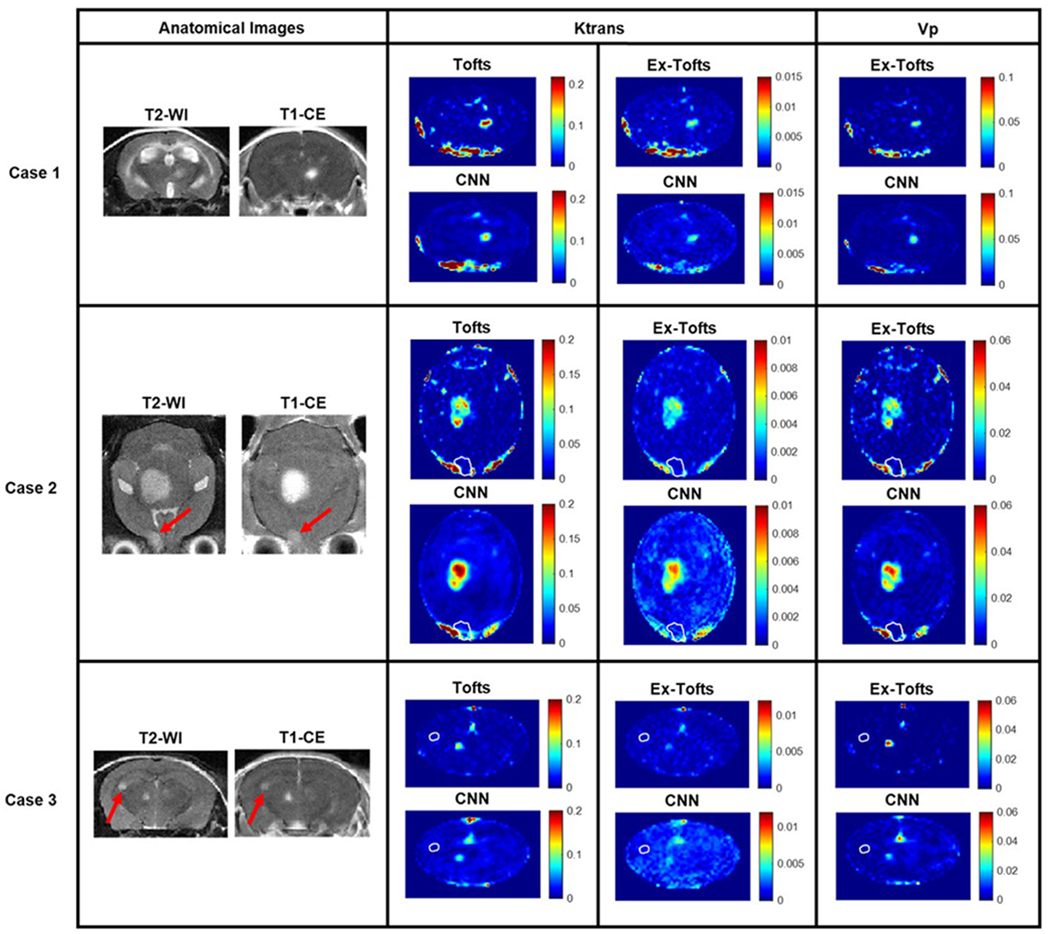
Brain metastasis transfer study. MR imaging investigation of the brain metastasis transfer study revealed lesions with differing size, shape, and permeability. Tofts Ktrans, Ex-Tofts Ktrans, and Ex-Tofts Vp target maps as well as their corresponding CNN PK maps were depicted. The T2-w detectable edge of the lesions in Case 2 and 3 that exhibited lower permeability (arrows) were superimposed as a white contour on the PK maps.

Following training and testing the proposed CNN with GBM, we performed a transfer study with brain metastasis to assess the potential of the network for alternative brain tumors. A total of 20 BCBM lesions were identified across the 15 dynamic slices used in this study. Unlike the glioma model, multiple brain metastases including both contrast enhanced and non-enhanced lesions were commonly seen in the BCBM model, representing an excellent phenotype for testing the CNN approach for translation. Three networks, one for each target PK parameter, were trained solely with GBM datasets followed by testing with BCBM. Fig. 4 depicts three representative cases from individual BCBM mice. A small lesion was identified for Case 1 on anatomical images that exhibited increased vascular permeability on the target Tofts and Ex-Tofts PK maps. The CNN PK maps retained the small size and shape of the BCBM lesion while learning each corresponding PK parameter intratumoral values. Anatomical images for Case 2 revealed two tumors, one of which had only minimal enhancement on the T_1_-CE image (Fig. 4, arrow). Target PK parameter maps displayed increased Ktrans and Vp values in the larger lesion, but lower values in the smaller lesion (arrow). In good agreement, the CNN PK maps revealed differential permeability between the two lesions. Similar to Case 2, Case 3 presented two lesions with differential vascular permeability, but with much smaller size than those of Case 2. Clearly, the CNN was able to recapitulate the small size and shape of the enhancing lesion and displayed low values of PK parameters in the minimally enhancing lesion.

**Fig. 5.**
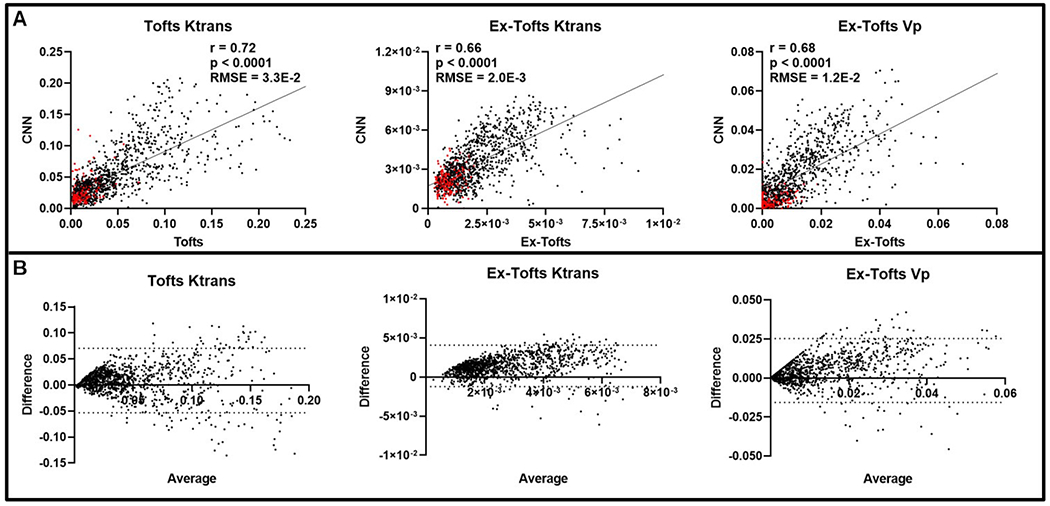
Intratumoral ensemble analysis of brain metastases. (A) Pixel-by-pixel data (n = 1148) of intratumoral regions were plotted that revealed significant linear correlations between the CNN and target PK parameters (*p* < 0.0001). Non-enhancing lesions (n = 187) Ktrans and Vp values are shown in red. RMSEs for all PK parameters were calculated and displayed. (B) Bland-Altman plots of PK parameters were generated with dashed lines corresponding to the upper and lower 95% confidence intervals.

Like the above glioma analysis, we conducted pixel-by-pixel comparisons for the BCBM. There were a total of 20 lesions identified across the DCE dynamic images (n = 15 slices), 14 of which were enhancing while six of them were non-enhancing, indicating an intact BBB. Intratumoral pixel-by-pixel comparison revealed significant linear correlation (*p* < 0.0001, n = 1148 pixels) for each of the three target PK parameters and their corresponding CNN PK values (Fig. 5A). The RMSE values for the Tofts Ktrans and the Ex-Tofts Vp were found to be smaller than the target PK parameter SDs, whereas the RMSE value for the Ex-Tofts Ktrans was found to be slightly larger than the SD (nRMSE = 1.43). Bland-Altman plots for all three PK parameters, particularly the Ex-Tofts Ktrans, exhibited a small positive bias (Fig. 5B).

## 4. Discussion

In this study, we have successfully developed a deep learning-based CNN model to generate the PK parameters for DCE MRI. CNNs are often implemented when working with images and are the most commonly used networks for pattern recognition tasks [36]. Hence, as generation of PK parameters is considered as a mapping problem to train a deep neural network to recognize patterns of increased heterogeneous intratumoral PK parameters from source DCE images, a CNN was chosen as an ideal network for this study. The specific CNN methodology as used in this study has previously been shown to yield accurate PK parameters directly from DCE images in different brain regions of stroke patients without conventional PK modeling. Hence, we hypothesized this CNN could be translated to small animal brain tumor research studies. Generative adversarial networks (GANs) have been gaining popularity in medical image processing as a more sophisticated deep learning method in which two networks compete against each other and hence might serve as a potential network for this application in future studies [36,37]. Nevertheless, our results show a good match for both Ktrans and Vp maps generated between the target PK parameter maps and the respective CNN PK parameter maps for brain tumors. Importantly, the CNN PK maps were shown to successfully recapitulate intratumoral heterogeneity and increased permeability in accordance with their target PK model parameter maps. The CNN approach to directly generate the PK maps from DCE dynamic images is time efficient (within a few seconds) and requires no human interference or additional data, whereas conventional PK modeling is much more time consuming and requires additional information including CA arrival, T_1_ mapping, and the AIF alongside complex curve-fitting algorithms.

K-fold cross validation is commonly implemented in deep learning applications to assess the performance of the neural network through cycling training and testing datasets [27,38,39]. This methodology ensures that the network can be transferred to alternative testing cases of variable phenotypes and that the network is avoiding overfitting of the training data through increasing the amount of testing data. Here we trained and tested a total of 21 networks, 18 for the GBM study and 3 for the brain metastasis transfer study. In the k-fold cross validation GBM study, similar RMSE and nRMSE values were found for each respective PK parameter (Tofts Ktrans, Ex-Tofts Ktrans, and Ex-Tofts Vp) across each network demonstrating the power of the deep learning approach to decipher intertumor variation for each testing case with high accuracy (Table 1). In particular, the target Ex-Tofts Ktrans and CNN produced consistently low RMSE values below the target parameter SD (nRMSE <1). Further quantification of the performance of these networks in the GBM study involved linear regression analysis for correlation and statistical significance, RMSE, and Bland-Altman plot analysis following intratumoral ensembles of the networks. For the GBM study, we found significant linear correlations (*p* < 0.0001), low RMSEs, and negligent over/under-prediction of the CNN for all three target PK parameters in both intratumoral and peripheral tumor regions (Fig. 3, Supplementary Fig. 2). Clearly the proposed CNN has the ability to generate PK parameter maps structurally similar to its target PK maps. Importantly, the CNN has the ability to be transferred not only between different PK parameters, but also between different PK models, i.e., the Tofts and Ex-Tofts models.

In line with the GBM study, significant linear correlations (*p* < 0.0001) were evidenced for all three PK parameters in the BCBM transfer study. The enhanced correlation coefficients for the BCBM transfer study might be a result of increasing the training datasets through the inclusion of an additional GBM imaging dataset. Hence, we anticipate that incorporation of additional training datasets will lead to more robust predictive capabilities of the network. Despite training the networks solely with large GBM images, the CNN was able to recapitulate the smaller size of BCBM tumors (Fig. 4). Moreover, the CNN accurately identified the PK parameter values in BCBMs with differential permeability as well as those with cerebrospinal fluid (CSF) enhancement (Fig. 4). In addition to intertumor variation, the CNN was capable of depicting intratumoral vascular heterogeneity in brain metastases as evidenced with the pixel-by-pixel analysis (Fig. 5) consistent with the finding in GBM (Fig. 3). Bland-Altman plot analysis revealed that the CNN on average might slightly over-predict the intratumoral PK values for the BCBM transfer study (Fig. 5). GBM is notoriously highly angiogenic with the presence of leaky microvessels and hence generate large PK permeability parameter values. On the contrary, brain metastases, particularly smaller lesions, often retain intact blood-tumor barriers (BTBs) and exhibit no enhancement on T_1_-CE images and low permeability on PK maps, as shown in Fig. 4 [32]. Hence, as we trained the networks with high permeability GBM datasets, we anticipate this to be a driving force for the slight CNN over-prediction of PK parameter values. Nevertheless, the results from the BCBM transfer study clearly indicate that the CNN has the ability to translate to alternative brain tumor phenotypes differing in size, shape, and/or permeability.

In general, deep learning and machine learning algorithms require huge amounts of data to perform efficiently upon training [40]. With limited availability of animal data in this study, there is a higher risk of overfitting the data, which can cause poor generalization and contribute to crude predictions. To solve such complications, the algorithm requires additional data to converge to a general case. Recent studies have shown the impact of augmented data in solving overfitting problems [41,42]. However, this approach of data augmentation was computationally costly and complex on this project as the DCE data trained was a complex time-dependent series of images. Hence, we proceeded with no additional data augmented to train the network. Despite a small number of training data for both the GBM study (n = 25 slices) and the brain metastasis transfer study (n = 30 slices), our results indicate that the proposed neural network can still efficiently recapitulate the tumor size, shape, and PK permeability parameters in both primary and metastatic brain tumors while avoiding overfitting of the data as evidenced through its translatability to alternative brain tumor phenotypes. This is likely attributable to the fact that each of these training ‘slices’ involved multiple complex dynamic images with thousands of pixels per image for the network to learn from.

## 5. Conclusions

In summary, we have successfully deployed the Tofts model and the Ex-Tofts model as well as a deep learning-based CNN to study vascular permeability in small animal brain tumors. Results and observations from the CNN infer that this deep learning approach could perform as precisely as the conventional PK models. Importantly, the CNN approach requires limited data without human intervention and can be completed with much less time. Future studies by training and testing the system with more data and including additional glioma models with differential phenotypes of tumor aggressiveness and angiogenesis will be needed to further establish its utility. Furthermore, implementation of an accurate AIF measurement in these small animals in a case-by-case basis prior to CNN training might enhance the accuracy of the produced conventional PK and CNN maps by taking into account individual variation. We anticipate that the proposed deep learning-based CNN will serve as a useful surrogate for small animal brain tumor research of vascular PK parameters.

## Supplementary Material

supplement Deep learning quantification of vascular pharmacokinetic parameters in mouse brain tumor models

## Figures and Tables

**Table 1.** Predictive performance of CNN in k-fold cross validation GBM study.

Target PK model	Metric	Network 1	Network 2	Network 3	Network 4	Network 5	Network 6
Tofts Ktrans	RMSE	8.60 × 10^−2^	8.73 × 10^−2^	2.37 × 10^−2^	4.34 × 10^−2^	1.34 × 10^−1^	7.05 × 10^−2^
	nRMSE	1.28	1.04	1.02	0.83	1.26	0.76

Ex-Tofts Ktrans	RMSE	1.87 × 10^−3^	2.24 × 10^−3^	1.50 × 10^−3^	1.88 × 10^−3^	1.84 × 10^−3^	2.31 × 10^−3^
	nRMSE	0.80	0.95	0.86	0.94	0.97	0.93

Ex-Tofts Vp	RMSE	1.72 × 10^−2^	2.36 × 10^−2^	1.58 × 10^−2^	1.88 × 10^−2^	2.04 × 10^−2^	2.27 × 10^−2^
	nRMSE	0.86	0.81	1.04	0.90	0.81	1.38

## Abbreviations

AIF: arterial input function
BBB: blood-brain barrier
BCBM: breast cancer brain metastasis
BTB: blood-tumor barrier
CA: contrast agent
CNN: convolutional neural network
CSF: cerebrospinal fluid
DCE MRI: dynamic contrast-enhanced MRI
DMEM: Dulbecco’s modified Eagle’s medium
EES: extravascular extracellular space
ETL: echo train length
Ex-Tofts: extended Tofts
FA: flip angle
FOV: field of view
GBM: glioblastoma multiforme
Kep: flux rate constant
Ktrans: volume transfer coefficient
nRMSE: normalized root mean squared error
NSA: number of scan averages
PK: pharmacokinetic
RARE: rapid imaging with refocused echoes
RMSE: root mean squared error
SD: standard distribution
SI: signal intensity
Sp: slope
ST: slice thickness
T_1_-w: T_1_-weighted
VA: variable flip angle
Ve: fractional volume of the EES
VIF: vascular input function
Vp: fractional volume of the blood plasma.
