# Supplementary material for: Deep learning quantification of vascular pharmacokinetic parameters in mouse brain tumor models
Source: Front Biosci (Landmark Ed). Author manuscript; Available in PMC 2022 Apr 28. (PMC9048985; doi:10.31083/j.fbl2703099)
Supplement: supplement Deep learning quantification of vascular pharmacokinetic parameters in mouse brain tumor models [file NIHMS1797007-supplement-supplement_Deep_learning_quantification_of_vascular_pharmacokinetic_parameters_in_mouse_brain_tumor_models.docx]

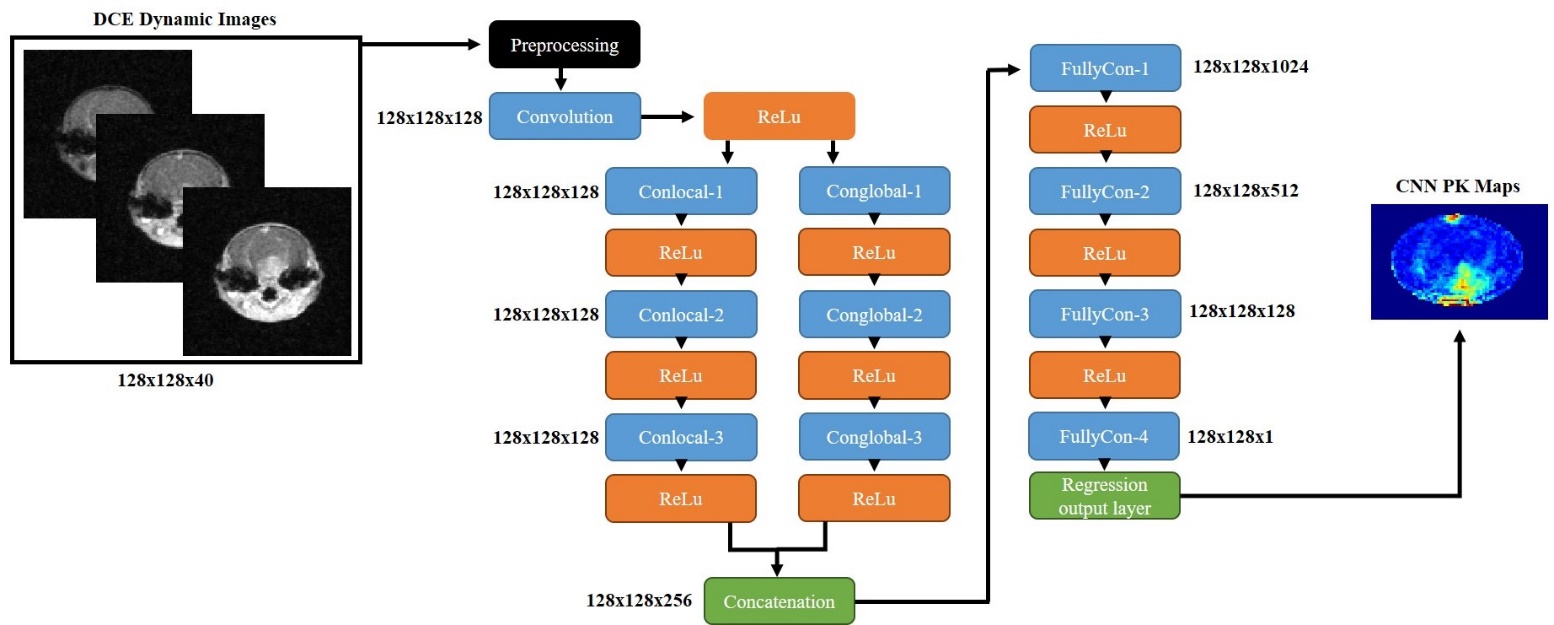


**Supplementary Fig. 1. Twenty four-layer CNN. The CNN was designed with two parallel pathways, global and local, to capture low-level and high-level features in the DCE time series.** All the convolutional layers are designed with 4 × 4 filters. DCE dynamic testing data can be fed into the pre-trained CNN to generate CNN PK maps.

Supplementary Table 1. Execution Time Profiles.

| Modeling Step | Tofts Model [s] | Ex-Tofts Model [s] | CNN [s] |
| --- | --- | --- | --- |
| CA arrival identification | <60 | <60 | N/A |
| T1 map MRI acquisition^1^ | 228 | 228 | N/A |
| T1 map generation | 3 | 3 | N/A |
| CA concentration map generation | 5 | 5 | N/A |
| PK parameter map generation | 15 | 245 | 2 |

^1^Additional MRI acquisitions (T1 variable flip angle images) required for T1 mapping for conventional PK models.


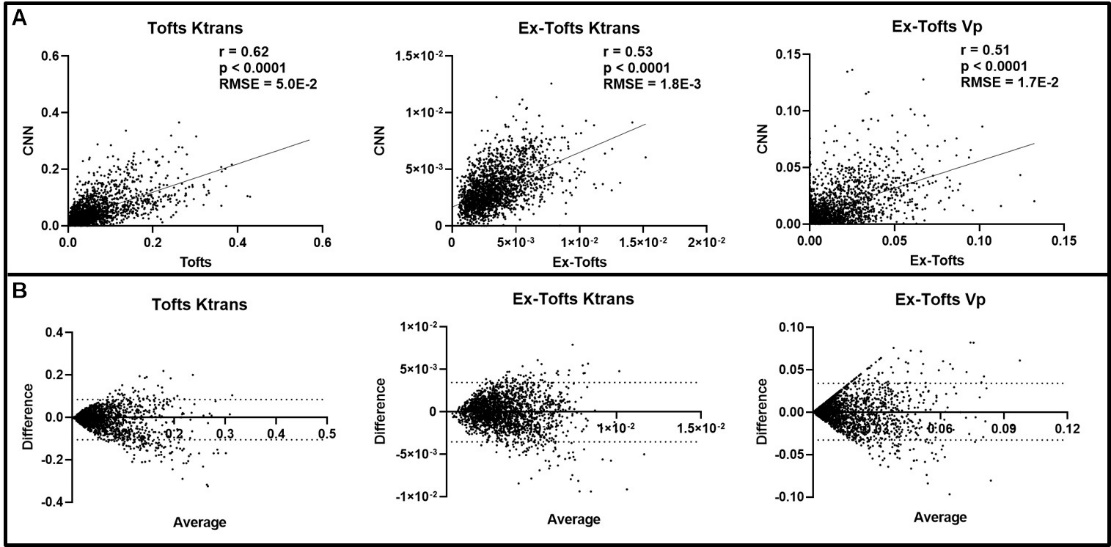


**Supplementary Fig. 2. Intratumoral ensemble analysis of peripheral GBM tissue.** (A) Pixel-by-pixel data (n = 2236) of tumor peripheral regions were plotted, revealing a significant linear correlation for Ktrans and Vp between CNN and target PK models (*p* < 0.0001). Low RMSEs were found between the target PK models and the CNN for all three parameters (RMSE < Target PK parameter SD). (B) Bland-Altman plots of PK parameters were generated with dashed lines corresponding to the upper and lower 95% confidence intervals.
